# Assessing the effects of partially substituting chicken breast meat with oyster mushroom stalk powder on the quality attributes of mushroom-chicken burgers

**DOI:** 10.1038/s41598-025-86127-3

**Published:** 2025-02-05

**Authors:** Sabah Mounir, Randa Mohamed, K. V. Sunooj, Sohier El-Saidy, Eman Farid

**Affiliations:** 1 Department of Food Science, Faculty of Agriculture, Zagazig University, Zagazig, 44519 Egypt; 2 Department of Food Science and Technology, Pondicherry University, Pondicherry, India

**Keywords:** Chicken burger, Oyster mushroom stalk powder, Chemical properties, Cooking quality, Color, Texture, Biochemistry, Chemical engineering

## Abstract

This study aimed to evaluate the effect of a partial substitution of chicken breast meat with oyster mushroom stalk powder on the quality attributes of mushroom-chicken burgers. Chicken breast meat was substituted with oyster mushroom stalk powder at different levels ranging from 2.5 to 10%, with an interval increase of 2.5%. The substitution level had a significant effect on the different characteristics studied. A decrease in both crude protein and crude lipid was observed as the substitution level increased from 2.5 to 10%. However, a contradictory trend was observed for crude fiber, ash, carbohydrates, antioxidant properties, water-binding capacity, and browning index. The cooking loss and shrinkage were reduced by about 81.6% and 72.6%, respectively, for the formulation prepared with 10% compared to the control sample. The formulation prepared with 2.5% showed the lowest hardness, chewiness, and shear force, which increased as the substitution level increased to 10%. The oyster mushroom stalk powder can be used at a level ranging from 2.5 to 5% to substitute chicken breast meat in the preparation of healthier and more functional chicken burgers.

**Supplementary Information:**

The online version contains supplementary material available at 10.1038/s41598-025-86127-3.

## Introduction

Animal-based foods, such as meat, play an important role in the human diet owing to their attractive sensorial characteristics and beneficial nutritional value (high-quality protein, minerals, and vitamins), while these foods are deficient in antioxidants, dietary fiber, and vitamin C^1^. On the other hand, the consumption of processed meat products, such as meat burgers, is associated with some chronic diseases, such as coronary heart disease, obesity, and cancer^2–4^, owing to the presence of saturated fatty acids. The demand to reduce the amount of animal proteins in the diet has grown due to the increased consumer’s awareness about healthier foods. Thus, the existing meat products can be reformulated to create healthier and more functional products^5^. In this context, the incorporation of plant-based products into animal-origin foods (meat products) has been applied to improve their nutritional profile (e.g., no cholesterol and low fat)^6^ and their functional properties, such as water retention and the ability to decrease cooking loss and to modify the texture. Mushrooms have been used as an alternative to animal proteins in the production of meat products due to their nutritive value, antioxidant properties, distinct flavor, and meat-like texture^7,8^. Previous studies have shown the potential use of mushrooms in meat-based products, such as chicken burgers, beef burgers, chicken sausages, and chicken frankfurters, to replace meat or fat and reduce lipid oxidation and sodium^9–13^. In this context, Rincón Soledad et al.^12^ investigated the effect of incorporating shiitake powder into chorizo sausages as a fat substitute at levels of 30, 40, 50, and 100%. The authors found that the sausage exhibited greater moisture, lower lipid content, and less cooking loss compared to the control samples, without affecting the texture. A similar trend was observed by Boylu et al.^13^ for the sausage prepared from pork meat partially substituted with fermented oyster mushrooms at levels of 25% and 50%.

On the other hand, there is an increasing interest in the valorization of plant by-products as functional ingredients in the food industry (i.e., meat-based products) because they are an excellent source of dietary fiber and bioactive compounds^14–16^. Similarly, mushroom by-products, e.g., mushroom stalks, are rich in dietary fiber and bioactive compounds^17,18^. Therefore, the valorization of mushroom stalks in different food preparations may contribute to reducing the environmental impact and producing healthier products with higher dietary fiber and antioxidants as well. Many studies have reported the successful applications of mushroom by-products (stems or stalks) or the extract of mushroom by-products in meat-based products (nuggets, burgers, and fermented sausages) or meat analogues^18–26^. These studies have highlighted the changes in the nutritional profile, enhanced cooking quality (increased cooking yield and reduced shrinkage) and moisture retention, and improved textural characteristics (reduced hardness and shear force) of the final products.

The literature available on substituting chicken breast meat with either mushrooms or mushroom by-products in the preparation of chicken meat products is too scant. Only one study was conducted by Banerjee et al.^18^ on the substitution of goat meat with enoki mushroom stem powder at a level ranging from 2 to 6%. Previous studies have reported the effect of a partial substitution of chicken meat with mushrooms on the nutritive value of the final products^19–21^. In general, the nutritional changes of these products were greatly dependent on the substitution level. A reduction in both protein and fat was observed with increasing the substitution level. While a contradictory trend was observed for the moisture content, fiber, and ash as the substitution level increased.

However, the higher fiber level may modify the texture of meat products. Banerjee et al.^18^ reported that the hardness, cohesiveness, and springiness of goat meat nuggets were slightly decreased as the substitution level with enoki mushroom stem powder increased from 2 to 6%. A similar trend was observed by Fu et al.^26^ for the sausages prepared from chicken breast meat substituted with mushroom at 30% and 50% compared to the control sample. Similarly, Wan Rosli et al.^20^ found that the texture parameters of the chicken patties decreased, except for the springiness that increased as the substitution level with grey oyster mushroom increased from 25 to 50%.

The water-binding capacity (WBC) is technologically important for developing and designing a new class of functional meat products, where the ability of meat to retain its own or added water can improve the cooking yield and textural characteristics. In this context, Rincón Soledad et al.^12^ stated that the chorizo sausages with shiitake powder had a lower cooking loss than the control sample due to shiitake powder’s high level of water retention. Likewise, Dosh et al.^22^ found that the WBC of chicken burgers increased while the cooking loss and shrinkage decreased as the substitution level with oyster mushroom powder increased from 10 to 15%.

As there are no studies reported on chicken burgers prepared from chicken breast meat substituted with oyster mushroom stalk powder, the objective of this study is to evaluate the effect of the substitution at different levels ranging from 2.5 to 10% on the nutritional and antioxidant properties, cooking quality, color, and textural characteristics of mushroom-chicken burgers.

## Results and discussion

### Chemical composition

Table 1 shows the chemical composition of the oyster mushroom stalk powder and the different cooked chicken burger samples. The results showed that the oyster mushroom stalk powder was rich in crude protein (25.56% db) and carbohydrates (58.60% db), while the moisture content, crude lipid, crude fiber, and ash were 14.57% (db), 0.95% (db), 7.98% (db), and 6.93% (db), respectively. These results are in line with those reported by Oluwafemi et al.^17^ and Banerjee et al.^18^. On the other hand, the cooked chicken burger (control sample) showed higher crude protein and crude lipid contents and lower contents of moisture, crude fiber, ash, and carbohydrates than the cooked mushroom-chicken samples. The contents of crude protein and crude lipid in the cooked control sample were found to be 79.36% and 11.32%, respectively, while those of the cooked mushroom-chicken burger samples were found in the range of 66.33–78.02% and 10.05–11.12%, respectively. This may be attributed to the different nutritional profile of oyster mushroom stalk powder compared to chicken breast meat. Besides, the inherent fibers of oyster mushroom stalk powder, which can bind more water and thus improve the water-binding capacity^16^. These fibers can retain the liquid containing minerals that may be released from the meat matrix during cooking, contributing to increasing the final moisture content and minerals. A gradual and significant (*P* ≤ 0.05) increase in the moisture content, crude fiber, ash, and carbohydrates was observed with increasing the substitution level. However, the crude protein and crude lipid showed a contradictory trend. Based on the obtained results, it could be said that substituting chicken breast meat with oyster mushroom stalk powder may modify the nutritional profile of cooked chicken burger samples, thus developing functional chicken burgers with higher health-promoting effects. The obtained results are in agreement with Banerjee et al.^18^, who found that the protein and fat content of the nuggets significantly decreased while the fiber content increased as the substitution level of goat meat with enoki mushroom stem powder increased from 2 to 6%. A similar trend was observed by Wan Rosli et al.^19,20^ and Yahya and Ting^16^ for chicken patties and chicken sausage prepared from chicken meat substituted at different levels with oyster mushroom.

**Table 1:** Chemical composition, phytochemicals, and antioxidant activity of oyster mushroom stalk powder and different cooked chicken burgers.

Response variable	Control	Cooked mushroom-chicken burger prepared from chicken breast meat partially substituted with oyster mushroom stalk powder at different levels				Oyster mushroom stalk powder
	0.00	2.5%	5%	7.5%	10%	
Moisture content (% db)	66.74 ± 0.15^e^	69.57 ± 0.11^d^	70.22 ± 0.08^c^	73.70 ± 0.13^b^	75.19 ± 0.14^a^	14.57 ± 0.13
Crude protein (% db)	79.36 ± 0.11^a^	78.02 ± 0.15^b^	72.93 ± 0.13^c^	69.90 ± 0.13^d^	66.33 ± 0.08^e^	25.56 ± 0.02
Crude lipid (% db)	11.32 ± 0.14^a^	11.12 ± 0.10^b^	11.04 ± 0.11^b^	10.79 ± 0.12^c^	10.05 ± 0.11^d^	0.95 ± 0.03
Crude fibre (% db)	1.51 ± 0.04^e^	1.81 ± 0.06^d^	2.07 ± 0.05^c^	2.18 ± 0.04^b^	2.53 ± 0.05^a^	7.98 ± 0.02
Ash (% db)	5.98 ± 0.06^c^	6.02 ± 0.02^c^	6.16 ± 0.04^b^	6.22 ± 0.08^ab^	6.27 ± 0.15^a^	6.93 ± 0.02
Cabohydrates (% db)	1.83 ± 0.12^e^	3.04 ± 0.03^d^	7.82 ± 0.04^c^	10.92 ± 0.04^b^	14.83 ± 0.00^a^	58.60 ± 0.09
TPC	2.609 ± 0.05^e^	3.193 ± 0.12^d^	4.122 ± 0.14^c^	4.531 ± 0.17^b^	6.513 ± 0.14^a^	9.545 ± 0.23
TFC	0.337 ± 0.06^e^	0.519 ± 0.05^d^	0.750 ± 0.05^c^	1.120 ± 0.08^b^	1.271 ± 0.03^a^	1.761 ± 0.07
Carotenoids (mg/100 g)	0.143 ± 0.06^e^	1.053 ± 0.06^d^	1.507 ± 0.04^c^	2.670 ± 0.04^b^	4.133 ± 0.05^a^	7.920 ± 0.16
AOA (%)	36.92 ± 0.14^e^	65.16 ± 0.18^d^	66.43 ± 0.16^c^	70.71 ± 0.13^b^	74.06 ± 0.18^a^	82.22 ± 0.13

Control: chicken burger prepared from 100% chicken breast meat; 2.5%: mushroom-chicken burger prepared from chicken breast meat substituted with mushroom stalk powder at 2.5%; 5%: mushroom-chicken burger prepared from chicken breast meat substituted with mushroom stalk powder at 5%; 7.5%: mushroom-chicken burger prepared from chicken breast meat substituted with mushroom stalk powder at 7.5%; 10%: mushroom-chicken burger prepared from chicken breast meat substituted with mushroom stalk powder at 10%; TPC: total phenolic content (mg GAE/g db); TFC: total flavonoid content (mg QE/g db).

Superscript letters in the same raw are statistically signifcantly diferent (*P* ≤ 0.05).

### Phytochemicals and antioxidant activity

Table 1 shows the phytochemicals (TPC, TFC, and carotenoids) and antioxidant activity of oyster mushroom stalk powder and the different chicken burger samples. The oyster mushroom stalk powder had a high antioxidant activity (82.22%), which is attributed to its inherent content of TPC (9.545 mg GAE/g db), carotenoids (7.920 mg/100 g), and TFC (1.761 mg QE/g db). Therefore, the cooked mushroom-chicken burgers showed higher levels of TPC, TFC, carotenoids, and AOA than the cooked control sample. Moreover, the TPC, TFC, carotenoids, and AOA of cooked mushroom-chicken burgers greatly depended on the substitution level. A gradual and significant increase in the TPC, TFC, carotenoids, and AOA was observed by increasing the substitution level. This may be attributed to the contribution of oyster mushroom stalk powder to increasing the TPC, TFC, and carotenoids, thus increasing the AOA.

The TPC, TFC, carotenoids, and AOA of the cooked mushroom-chicken burgers were in the range of 3.193–6.513 mg GAE/g db, 0.519–1.271 mg QE/g db, 1.053–4.133 mg/100 g, and 65.16–74.06%, respectively. Whereas those of the cooked control sample were found to be 2.609 mg GAE/g db, 0.337 mg QE/g db, 0.143 mg/100 g, and 36.92%, respectively (Table 1).

The obtained results are in agreement with those obtained by Banerjee et al.^18^, who found that the TPC of the goat nuggets significantly increased as the substitution level of goat meat with enoki mushroom stem powder increased from 2 to 6%.

### Water-binding capacity and cooking quality

Table 2 shows the water binding capacity (WBC) of the oyster mushroom stalk powder and different chicken burger samples. The results showed that the raw mushroom-chicken burgers had a higher WBC than the raw control sample, which increased as the substitution level increased. The WBC of the raw mushroom-chicken burgers was in the range of 61.46–79.83%, while that of the raw control sample was 54.42%. This may be attributed to many factors, including (1) the higher ability of the inherent fiber of mushroom stalk powder to retain more liquid^27^; (2) the lower fat content in mushroom-chicken burgers, which makes proteins bind freely to water molecules, thereby increasing the WBC^28^; and (3) the formation of a complex three-dimensional gel network due to the interaction between non-animal (the protein of oyster mushroom stalk powder) and animal proteins (the protein of chicken breast meat), which can retain more liquid^29^.

**Table 2:** Water-binding capacity, cooking quality, and color characteristics of different chicken burgers.

Response variable	Control	Mushroom-chicken burger prepared from chicken breast meat partially substituted with oyster mushroom stalk powder at different levels				Oyster mushroom stalk powder
	0.00	2.5%	5%	7.5%	10%	
WBC (%)	54.42 ± 0.15^e^	61.46 ± 0.13^d^	74.18 ± 0.10^c^	77.14 ± 0.11^b^	79.83 ± 0.15^a^	633.2 ± 1.55
Cooking loss (%)	31.06 ± 0.26^a^	22.95 ± 0.20^b^	18.94 ± 0.18^c^	10.89 ± 0.15^d^	5.70 ± 0.10^e^	ND
Shrinkage (%)	25.62 ± 0.31^a^	17.25 ± 0.23^b^	13.96 ± 0.18^c^	7.92 ± 0.09^d^	7.03 ± 0.06^e^	ND
L*	49.72 ± 0.06^a^	47.39 ± 0.10^b^	44.25 ± 0.08^c^	41.02 ± 0.04^d^	40.45 ± 0.02^e^	41.89 ± 0.05
a*	2.22 ± 0.08^e^	2.45 ± 0.08^d^	2.56 ± 0.06^c^	3.02 ± 0.03^b^	3.10 ± 0.04^a^	4.21 ± 0.03
b*	7.25 ± 0.04^e^	7.57 ± 0.06^d^	7.93 ± 0.04^c^	8.47 ± 0.02^b^	9.68 ± 0.04^a^	17.28 ± 0.06
ΔE	0.00^e^	2.37 ± 0.02^d^	5.43 ± 0.02^c^	8.63 ± 0.02^b^	9.72 ± 0.04^a^	ND
BI	18.70 ± 0.04^e^	20.85 ± 0.23^d^	23.64 ± 0.16^c^	27.96 ± 0.04^b^	32.60 ± 0.05^a^	59.39 ± 0.18

Control: chicken burger prepared from 100% chicken breast meat; 2.5%: mushroom-chicken burger prepared from chicken breast meat substituted with mushroom stalk powder at 2.5%; 5%: mushroom-chicken burger prepared from chicken breast meat substituted with mushroom stalk powder at 5%; 7.5%: mushroom-chicken burger prepared from chicken breast meat substituted with mushroom stalk powder at 7.5%; 10%: mushroom-chicken burger prepared from chicken breast meat substituted with mushroom stalk powder at 10%; ; WBC: water-binding capacity; L*: color brightness (0: black, 100: white); a* (-a*: greenness, +a*: redness); b* (-b*: blueness, +b*: yellowness); ΔE: total color change; BI: browning index; ND: not determined.

Superscript letters in the same raw are statistically signifcantly diferent (*P* ≤ 0.05).

**Fig. 1:**
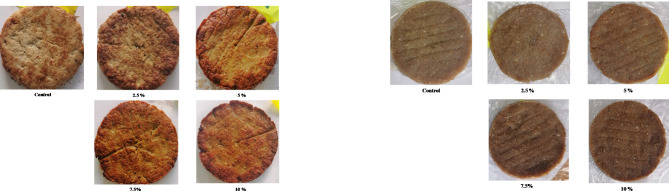
Visual appearance of different chicken burger formulations; right: raw burger samples; left: cooked burger samples.

The control sample showed higher cooking loss and shrinkage than the mushroom-chicken burgers, being 31.06% and 25.62% versus 5.70–22.95% and 7.03–17.25%, respectively (Table 1). Moreover, a gradual and significant decrease in both the cooking loss and shrinkage was observed with increasing the substitution level; the cooking loss and shrinkage were reduced by about 81.6% and 72.6%, respectively, for the formulation prepared with 10% oyster mushroom stalk powder, compared to the control sample. This may be attributed to the gradual improvement in the WBC of mushroom-chicken burgers, which depends on the fiber content. The higher cooking loss and shrinkage of the control sample may be attributed to the increased extent of protein denaturation, collagen shrinkage, and the collapse of the protein matrix^30,31^, which in turn disables the protein to bind more liquid, thus increasing the cooking loss and shrinkage^28,32^. However, the improved cooking quality of the mushroom-chicken burger samples may be due to (1) the lower protein denaturation extent during cooking due to the lower animal protein present in these samples compared to the control sample, presence of denatured proteins (the proteins of oyster mushroom stalk powder were already denatured during drying), and preventing the collapse of protein matrix due to the presence of fiber^33^; (2) the higher WBC due to the presence of fiber with a higher level; and (3) the formation of a firmer and more compact structure^34^, resulting from the interaction between non-animal and animal proteins, which can retain more water^29^, thereby reducing the cooking loss and shrinkage during cooking. Therefore, substituting chicken breast meat with oyster mushroom stalk powder can help maintain the burger’s shape, structure, and integrity during processing, storage, and consumption (Fig. 1) and reduce syneresis during the cooking process, increasing the cooking yield of chicken burgers^35^.

The obtained results are in agreement with those obtained by Dosh et al.^22^, who reported that the WBC of chicken burgers significantly increased while the cooking loss and shrinkage decreased as the substitution level of chicken meat with oyster mushrooms increased from 10 to 15%. Similarly, Banerjee et al.^18^ found that the cooking loss of the goat meat nuggets significantly decreased as the substituting level of goat meat with enoki mushroom stem powder increased from 2 to 6%.

### Color parameters

Table 2 shows the color parameters of oyster mushroom stalk powder and the different chicken burger samples. The L*, a*, and b* of oyster mushroom stalk powder were found to be 41.89, 4.21, and 17.28, respectively, with a browning index (BI) of 59.39 (Table 2), indicating the brownish color of oyster mushroom stalk powder that may be attributed to the presence of melanin pigments^36^ and the formation of the Maillard reaction during drying^37^.

The raw control sample was lighter in color than the raw mushroom-chicken burgers, with an L* value of 49.72 versus 40.45–47.39, respectively. The values of a* and b* of the raw control sample were found to be 2.22 and 7.25, respectively, whereas those of raw mushroom-chicken burgers were found in the range of 2.45–3.10 and 7.57–9.68, respectively. The total color change (*ΔE*) for the raw mushroom-chicken burgers varied from 2.37 to 9.72, while the browning index (BI) was in the range 20.85–32.60 versus 18.70 for the raw control sample.

Furthermore, a significant decrease in the color lightness was observed with increasing the substitution level, while the color redness and yellowness showed a contradictory trend. These differences may be attributed to the brownish color of oyster mushroom stalk powder and its inherent content of carotenoids^38^. Additionally, the lower L* and increased a* and b* may contribute to increasing the BI and *ΔE* of the raw mushroom-chicken burgers. A similar trend was observed by Wan Rosli et al.^20^; the L* value of chicken patties decreased while the a* value increased as the substitution level with the grey oyster mushroom increased from 25 to 50%.

### Textural characteristics

Table 3 shows the textural characteristics of the different raw and cooked chicken burger samples. It was found that substituting chicken breast meat with oyster mushroom stalk powder may modify the textural characteristics of raw and cooked mushroom-chicken burgers. The raw control sample had significantly higher hardness (11.71 N) than the raw mushroom-chicken burgers (5.48–11.41 N). However, the raw control sample had lower adhesiveness, cohesiveness, and springiness compared to the raw mushroom-chicken burgers. It is worthwhile noting that the mushroom-chicken burger sample prepared with 2.5% as a substitution level showed the lowest chewiness value compared to the other samples. The textural changes may be associated with the variations in the levels of WBC and fiber in both the control sample and mushroom-chicken burgers.

**Table 3:** Textural characteristics of different raw and cooked chicken burgers.

Response variable	Raw control	Raw mushroom-chicken burger prepared from chicken breast meat partially substituted with oyster mushroom stalk powder at different levels			
	0.00	2.5%	5%	7.5%	10%
Hardness (N)	11.71 ± 0.10^a^	5.48 ± 0.15^e^	9.25 ± 0.17^d^	9.96 ± 0.12^c^	11.41 ± 0.08^b^
Adhesiveness (g.cm)	67.0 ± 0.12^e^	87.0 ± 1.41^d^	162.0 ± 2.12^c^	225.0 ± 2.83^b^	310.0 ± 2.12^a^
Cohesiveness	0.36 ± 0.01^d^	0.46 ± 0.03^c^	0.59 ± 0.04^b^	0.78 ± 0.05^a^	0.75 ± 0.02^a^
Springiness (mm)	4.02 ± 0.06^e^	5.73 ± 0.06^d^	6.97 ± 0.09^c^	8.76 ± 0.08^b^	15.31 ± 0.05^a^
Chewiness (g.cm)	172.0 ± 1.41^d^	149.0 ± 2.10^e^	389.0 ± 8.49^c^	696.0 ± 5.66^b^	1345.0 ± 4.95^a^

	Cooked control	Cooked mushroom-chicken burger prepared from chicken breast meat partially substituted with oyster mushroom stalk powder at different levels			
	0.00	2.5%	5%	5%	10%
Shear force (N)	16.48 ± 0.18^c^	14.54 ± 0.01^e^	16.25 ± 3.27^d^	16.74 ± 0.84^d^	18.23 ± 0.06^a^

Control: chicken burger prepared from 100% chicken breast meat without mushroom stalk powder; 2.5%: mushroom-chicken burger prepared from chicken breast meat substituted with mushroom stalk powder at 2.5%; 5%: mushroom-chicken burger prepared from chicken breast meat substituted with mushroom stalk powder at 5%; 7.5%: mushroom-chicken burger prepared from chicken breast meat substituted with mushroom stalk powder at 7.5%; 10%: mushroom-chicken burger prepared from chicken breast meat substituted with mushroom stalk powder at 10%.

Superscript letters in the same raw are statistically signifcantly diferent (*P* ≤ 0.05).

On the other hand, a gradual and significant increase in the textural characteristics studied was observed with increasing the substitution level from 2.5 to 10%. This may be due to the gradual increase in the fiber levels in the formulations, which are provided by the mushroom stalk powder. The protein-fiber interactions and the avoiding of the new rearrangements of liaison protein-fiber may cause an increase in the hardness of the mushroom-chicken burgers^39^ by imparting more resistance to deformation^40^. Additionally, the water that acts as a plasticizer may contribute to increasing the elasticity (rubbery matrix), which in turn increases the elastic resistance, cohesiveness, and springiness. Besides, the presence of a higher level of chewy, insoluble fiber provided by the oyster mushroom stalk powder may contribute to increasing the chewability of the samples.

Similar to the hardness, mushroom-chicken burgers showed lower shear force than the control sample, except for the formulations prepared with oyster mushroom stalk powder at 7.5% and 10%. The shear force of the cooked control sample was 16.48 N, while that of the cooked mushroom-chicken burgers ranged between 14.54 and 18.23 N, which was significantly affected by the substitution level. The increased shear force of the cooked control sample may be attributed to the increased texture’s toughness^41^. During Cooking, the high extent of protein denaturation, the collagen shrinkage, and the collapse of the protein matrix^30,31^ lead to forming a dense protein network, and thus the texture becomes more tough^32^. But the decreased shear force of the mushroom-chicken samples, except for the formulation prepared with 7.5% and 10% oyster mushroom stalk powder, may be attributed to the combined effect of the increased WBC, the cooking softening^42^, and the breaking down of the insoluble fiber. However, it is noteworthy that increasing the WBC at certain levels may cause the texture to become very elastic, thus increasing the shear force, as in the case of mushroom-chicken burgers prepared with 7.5% and 10%., The increase in the fiber content is another supporting explanation.

The obtained results are in agreement with Lee et al.^43^, who found that the hardness and chewiness of breakfast sausage containing kimchi powder were higher than the control sample. Similarly, Confrades et al.^44^ found that the hardness and chewiness of bologna sausage increased with increasing the fiber content (e.g., soy fiber). On the other hand, Shen et al.^41^ also found that the shear force of beef patties increased as the PPI (pea protein isolate) level increased from 2.5 to 5%. However, Wan Rosli et al.^20^ found that the hardness, cohesiveness, and chewiness of chicken patties decreased, while springiness increased as the substitution level with grey oyster mushroom increased from 25 to 50%.

### Pearson correlation

Some correlations were found between the substitution level and the different response variables, which showed the predominant effect of the substitution level and WBC on the responses studied (Table 4), including (1) a negative correlation was found between the substitution level and both the crude protein and crude lipid, while a direct correlation was observed with the crude fiber and carbohydrates, indicating the modification of nutritional profile due to the chemical profile of oyster mushroom stalk powder; (2) a positive correlation was found between the substitution level and the antioxidant properties, reflecting the richness of oyster mushroom stalk powder in TPC, TFC, and carotenoids, which had a higher antioxidant properties; (3) a direct positive correlation was observed between the substitution level and the BI and ΔE, showing the effect of brownish color of oyster mushroom stalk powder on the L*, a*, and b*; (4) an inverse correlation was observed between the substitution level and both the cooking loss and shrinkage, while a direct correlation was found with the WBC, showing the higher ability of inherent fiber of oyster mushroom stalk powder to retain and bind the meat’s or added water; (5) a positive correlation was found between the substitution level and the textural characteristics, indicating the effect of fiber and non-meat protein on the texture alteration; (6) a negative correlation was found between the WBC and crude protein, reflecting the lower WBC of non-animal proteins (inherent proteins of oyster mushroom stalk powder) compared to the animal proteins (chicken breast meat), which was compensated with increasing the fiber level; and (7) a direct correlation was observed between the WBC and the textural characteristics, indicating the texture modification (rubbery matrix) due to the water plasticizer effect.

**Table 4:** Correlation between the substitution level of chicken breast meat with oyster mushroom stalk powder and the various characteristics of mushroom-chicken burger formulations.

*r*	*R*	MC	Protein	Ash	Fibre	Lipid	Carb	TPC	TFC	Carot.	AOA	CL	SH	L*	a*	b*	ΔE	BI	H	AD	CO	SP	CH	SF	WBC
R	1	0.97	-0.99	0.96	0.98	-0.92	0.99	0.96	0.99	0.98	0.98	-0.99	-0.97	-0.97	0.96	0.96	0.98	0.99	0.94	0.99	0.92	0.92	0.97	0.98	0.92
MC	0.97	1	-0.94	0.89	0.92	-0.90	0.95	0.90	0.98	0.97	0.99	-0.99	-0.97	-0.94	0.99	0.94	0.96	0.98	0.85	0.96	0.92	0.90	0.95	0.90	0.83
Protein	-0.99	-0.94	1	-0.99	-0.98	0.88	-0.99	-0.94	-0.98	-0.95	-0.95	0.97	0.96	0.97	-0.93	-0.94	-0.98	-0.98	-0.98	-0.99	-0.92	-0.89	-0.95	-0.99	-0.96
Ash	0.96	0.89	-0.99	1	0.94	-0.79	0.98	0.89	0.96	0.89	0.90	-0.94	-0.95	-0.98	0.90	0.87	0.98	0.93	0.99	0.96	0.94	0.81	0.89	0.97	0.99
Fibre	0.98	0.92	-0.98	0.94	1	-0.95	0.99	0.99	0.94	0.97	0.95	-0.96	-0.90	-0.91	0.89	0.98	0.94	0.98	0.94	0.99	0.84	0.96	0.98	0.99	0.90
Lipid	-0.92	-0.90	0.88	-0.79	-0.95	1	-0.90	-0.98	-0.87	-0.97	-0.95	0.92	0.81	0.79	-0.84	-0.99	-0.83	-0.95	-0.78	-0.93	-0.70	-0.99	-0.99	-0.90	-0.72
Carb	0.99	0.95	-0.99	0.98	0.99	-0.90	1	0.96	0.98	0.96	0.96	-0.98	-0.96	-0.97	0.94	0.95	0.98	0.98	0.97	0.99	0.92	0.91	0.96	0.99	0.95
TPC	0.96	0.90	-0.94	0.89	0.99	-0.98	0.96	1	0.91	0.97	0.95	-0.94	-0.86	-0.85	0.85	0.99	0.89	0.97	0.88	0.97	0.77	0.99	0.99	0.97	0.83
TFC	0.99	0.98	-0.98	0.96	0.94	-0.87	0.98	0.91	1	0.96	0.98	-0.99	-0.99	-0.99	0.99	0.92	0.99	0.98	0.93	0.98	0.96	0.87	0.94	0.95	0.92
Carot.	0.98	0.97	-0.95	0.89	0.97	-0.97	0.96	0.97	0.96	1	0.99	-0.98	-0.92	-0.90	0.94	0.99	0.93	0.99	0.86	0.98	0.84	0.97	0.99	0.94	0.82
AOA	0.98	0.99	-0.95	0.90	0.95	-0.95	0.96	0.95	0.98	0.99	1	-0.99	-0.95	-0.93	0.97	0.97	0.95	0.99	0.86	0.98	0.89	0.94	0.98	0.93	0.83
CL	-0.99	-0.99	0.97	-0.94	-0.96	0.92	-0.98	-0.94	-0.99	-0.98	-0.99	1	0.98	0.96	-0.98	-0.96	-0.98	-0.99	-0.90	-0.99	-0.93	-0.92	-0.97	-0.95	-0.88
SH	-0.97	-0.97	0.96	-0.95	-0.90	0.81	-0.96	-0.86	-0.99	-0.92	-0.95	0.98	1	0.99	-0.99	-0.88	-0.99	-0.95	-0.91	-0.95	-0.98	-0.82	-0.89	-0.92	-0.91
L*	-0.97	-0.94	0.97	-0.98	-0.91	0.79	-0.97	-0.85	-0.99	-0.90	-0.93	0.96	0.99	1	-0.96	-0.86	-0.99	-0.94	-0.95	-0.95	-0.99	-0.80	-0.88	-0.94	-0.96
a*	0.96	0.99	-0.93	0.90	0.89	-0.84	0.94	0.85	0.99	0.94	0.97	-0.98	-0.99	-0.96	1	0.89	0.97	0.96	0.85	0.94	0.96	0.84	0.90	0.88	0.85
b*	0.96	0.94	-0.94	0.87	0.98	-0.99	0.95	0.99	0.92	0.99	0.97	-0.96	-0.88	-0.86	0.89	1	0.89	0.98	0.85	0.97	0.78	0.99	0.99	0.95	0.80
ΔE	0.98	0.96	-0.98	0.98	0.94	-0.83	0.98	0.89	0.99	0.93	0.95	-0.98	-0.99	-0.99	0.97	0.89	1	0.96	0.96	0.97	0.98	0.83	0.91	0.95	0.95
BI	0.99	0.98	-0.98	0.93	0.98	-0.95	0.98	0.97	0.98	0.99	0.99	-0.99	-0.95	-0.94	0.96	0.98	0.96	1	0.91	0.99	0.89	0.95	0.99	0.97	0.88
H	0.94	0.85	-0.98	0.99	0.94	-0.78	0.97	0.88	0.93	0.86	0.86	-0.90	-0.91	-0.95	0.85	0.85	0.96	0.91	1	0.94	0.90	0.80	0.87	0.97	0.99
AD	0.99	0.96	-0.99	0.96	0.99	-0.93	0.99	0.97	0.98	0.98	0.98	-0.99	-0.95	-0.95	0.94	0.97	0.97	0.99	0.94	1	0.90	0.94	0.98	0.99	0.92
CO	0.92	0.92	-0.92	0.94	0.84	-0.70	0.92	0.77	0.96	0.84	0.89	-0.93	-0.98	-0.99	0.96	0.78	0.98	0.89	0.90	0.90	1	0.71	0.81	0.87	0.92
SP	0.92	0.90	-0.89	0.81	0.96	-0.99	0.91	0.99	0.87	0.97	0.94	-0.92	-0.82	-0.80	0.84	0.99	0.83	0.95	0.80	0.94	0.71	1	0.99	0.92	0.74
CH	0.97	0.95	-0.95	0.89	0.98	-0.99	0.96	0.99	0.94	0.99	0.98	-0.97	-0.89	-0.88	0.90	0.99	0.91	0.99	0.87	0.98	0.81	0.99	1	0.96	0.82
SF	0.98	0.90	-0.99	0.97	0.99	-0.90	0.99	0.97	0.95	0.94	0.93	-0.95	-0.92	-0.94	0.88	0.95	0.95	0.97	0.87	0.99	0.87	0.92	0.96	1	0.95
WBC	0.92	0.83	-0.96	0.99	0.90	-0.72	0.95	0.83	0.92	0.82	0.83	-0.88	-0.91	-0.96	0.85	0.80	0.95	0.88	0.99	0.92	0.92	0.74	0.82	0.95	1

r: correlation coefficient; R: substitution level of chicken breast meat with oyster mushroom stalk powder; MC: moisture content (% db); protein: crude protein (% db); Fibre: crude fibre (% db); Lipid: crude lipid (% db); Carb: carbohydrate content (% db); TPC: total phenolic content (mg GAE/g db); TFC: total flavonoid content (mg QE/g db); Carot: carotenoids (mg/100 g); AOA: antioxidant activity (%); CL: cooking loss(%); SH: shrinkage (%); L*: color brightness (0: black, 100: white); a* (-a*: greenness, +a*: redness); b* (-b*: blueness, +b*: yellowness); ΔE: total color change; BI: browning index; H: hardness (N); AD: adhesiveness (g.cm); CO: cohesiveness, SP: springiness (mm); CH: chewiness (g.cm); SF: shear force (N); WBC: water-binding capacity (%).

## Conclusion

This study contributes to increasing knowledge of hybrid foods to meet the dietary preferences of consumers. The results showed that the changes in the chemical composition, color, and textural characteristics of mushroom-chicken burgers were significantly dependent on the substitution level. An improvement in the WBC, cooking quality, and antioxidant properties was observed as the substitution level increased. This study highlighted the impact of fiber content and WBC on the cooking quality and textural properties of mushroom-chicken burgers. The formulations prepared with 2.5% oyster mushroom stalk powder showed the lowest hardness, chewiness, and shear force, which increased as the substitution level increased. This work demonstrated the possibility of valorizing the oyster mushroom stalk powder in the preparation of healthier and more functional chicken burgers by substituting the chicken breast meat at a level of 2.5–5%. Although the findings are promising, a study of microbiological quality and storage stability during different periods of frozen storage at -18 °C is necessary, which is suggested in the future work. Besides, a study of the consumer acceptance and economic feasibility is also recommended.

## Materials and methods

### Raw materials

Fresh chicken breast meat and other ingredients (fresh onion, eggs, and spices) were purchased from the local market (Zagazig, Egypt), while the oyster mushroom stalk powder was obtained from the Agricultural Research Centre (Giza, Egypt).

### Preparation of chicken burger formulations

**Fig. 2:**
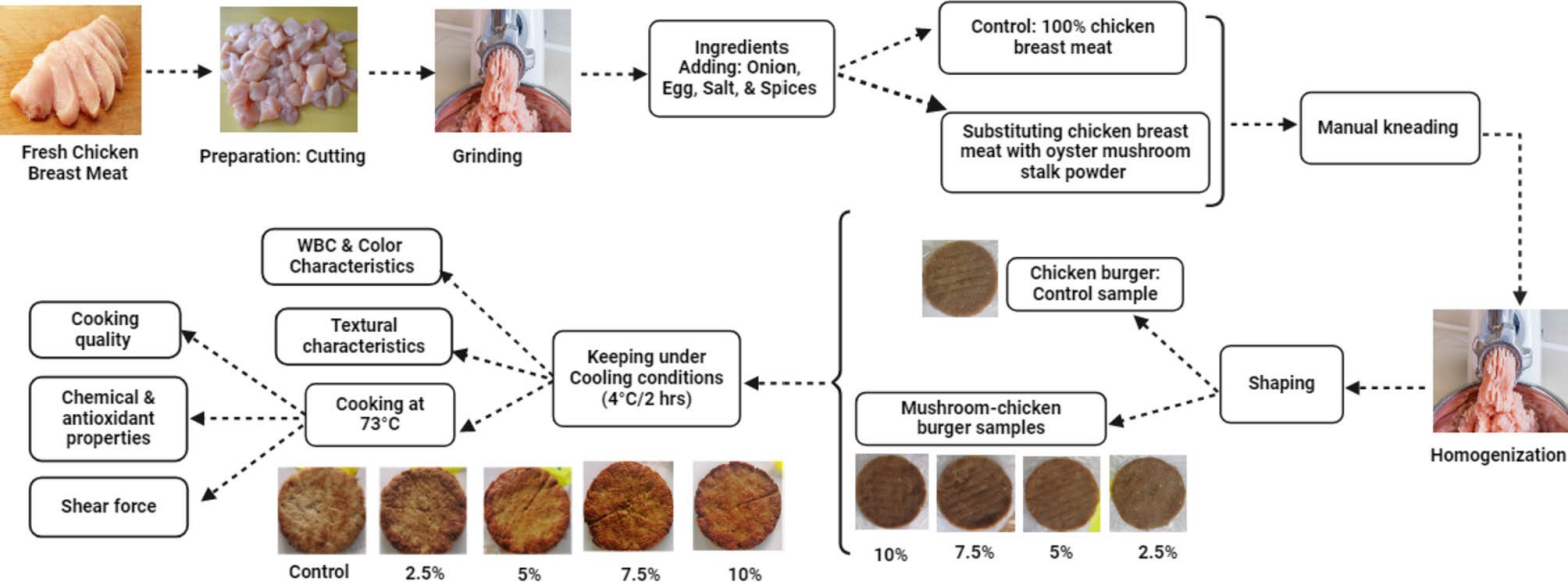
Protocol adopted in the preparation of different chicken burger formulations.

The fresh chicken breast meat was manually cut into small pieces and ground twice using a kitchen meat grinder (Tornado Meat Grinder 2000 W, Stainless Discs, Turbo Speed, White MG-2000, China). About 20 g of finely ground onion, one whole egg, 3 g of salt, and 5 g of a spice mixture (black pepper, cumin, cinnamon, ginger, sumac, nutmeg, cardamom, Lowry, Chinese cubeb, and clove) were added to 500 g of ground chicken breast meat. For the mushroom-chicken burgers, four formulations were prepared by substituting chicken breast meat with oyster mushroom stalk powder at different levels ranging from 2.5 to 10%, with an interval increase of 2.5% (Table S1). These ratios were determined on the basis of 100 g of minced chicken breast meat. The blend was first manually kneaded, homogenized using the same grinder, and then shaped with a stainless steel burger press (1.2 ± 0.2 cm thickness and 11.2 ± 0.3 cm diameter). The different samples were kept under cooling conditions (4 ± 2 °C) for 2 h and then cooked using a preheated grill brushed with a small amount of refined vegetable oil until the internal temperature reached 73 ± 2 °C (Fig. 2).

### Assessments and characterizations

#### Chemical composition

The moisture content, crude protein, crude lipid, crude fiber, and ash of the oyster mushroom stalk powder and different cooked chicken burger formulations were determined on a dry basis, according to AOAC^45^. The moisture content and carbohydrates were calculated using Eqs. (1) and (2), as follows:

1 $$\:Moisture\:content\:\left(\%\:db\right)=\frac{{W}_{1}-{W}_{2}}{{W}_{2}}$$

Where: W_1_ and W_2_ are the weights of the fresh sample and the sample after drying, respectively.

2 $$\:Carbohydrates\:\left(\%\:db\right)=100\:\left(\%\:crude\:protein+\%\:crude\:lipid+\%\:ash+\%\:crude\:fiber\right)$$

#### Phytochemicals and antioxidant activity

About 10 g of the sample was extracted in 100 mL of methanol, followed by filtration through Whatman No. 1 filter paper. The residue was re-extracted with another 60 mL of methanol and filtered through Whatman No. 1 filter paper. The filtrate was evaporated by a rotary vacuum evaporator at 40 °C (Buchi Rota-vapor R-124 Rotary Evaporator, Switzerland).

The total phenolic content (TPC) of the methanolic extract was determined by the Folin-Ciocalteu assay using gallic acid as a standard according to Behbahani^46^, with some modifications. About 250 µL of the methanolic extract was diluted with 3 mL of distilled water, followed by adding 0.5 mL of Folin-Ciocalteu reagent. The mixture was then homogenized for 3 min using a vortex, followed by adding 2 mL of Na_2_CO_3_ solution (1 M). The mixture was kept at room temperature (32 ± 2 °C) for 1 h in the dark. The absorbance was read at 765 nm using a UV-VIS spectrophotometer (DU 800; Beckman Coulter, Fullerton, CA, USA) against a blank sample containing distilled water. The TPC was expressed as mg GAE/g db using the calibration curve.

The total flavonoid content (TFC) of the methanolic extract was quantified by the aluminum chloride colorimetric method using quercetin as a standard, according to Chang et al.^47^. About 50 µL of the methanolic extract was brought to 1 mL with methanol, and 4 mL of distilled water and 0.3 mL of a 5% NaNO_2_ solution were then added. The mixture was perfectly mixed and allowed to stand for 5 min at room temperature (32 ± 2 °C), followed by adding 3 mL of a 10% AlCl_3_ solution. Subsequently, the mixture was allowed to stand for a further 6 min at room temperature, followed by adding 2 mL of 1 M NaOH. The final volume was made up to 10 mL with distilled water and then thoroughly mixed. The final mixture was allowed to stand for 15 min at room temperature, and absorbance was measured at 510 nm by a UV-VIS spectrophotometer (DU 800; Beckman Coulter, Fullerton, CA, USA) against the blank (without the sample). The total flavonoid content was calculated and expressed as mg QE/g db using the calibration curve.

The carotenoids were determined according to Nagata and Yamashita^48^, with slight modifications. About 150 mg of the sample was perfectly shaken with 10 mL of 80% acetone and allowed to stand for at least 16 h at room temperature (32 ± 2 °C) in darkness. This mixture was then filtered through Whatman No. 1 filter paper, followed by adding acetone to make the final volume 100 mL with acetone. The absorbance was measured at 440 nm against the blank (acetone), and the carotenoids were calculated using Eq. (3).

3 $$\:Carotenoids\:(mg/100\:g)\:={Abs}_{440}\:\times\:4.695$$

The antioxidant activity was determined according to Brand-Williams et al.^49^ by the DPPH (2, 2-diphenyl-1-picrylhydrazyl) assay. A mixture containing 1 g of sample and 50 mL of absolute ethanol (80%) was heated in a water bath at 70 °C for 1 h, filtered by Whatman No. 1 filter paper, and the filtrate was then evaporated at room temperature (32 ± 2 °C). 0.5 mL of the extract was added to 3 mL of absolute ethanol, followed by 0.3 mL of a DPPH radical solution (0.5 mM). The mixture was then kept at room temperature for 100 min of reaction, and the absorbance was subsequently read at 517 nm by a UV-VIS spectrophotometer (DU 800; Beckman Coulter, Fullerton, CA, USA). A blank solution was prepared by mixing 3.3 mL of absolute ethanol and 0.5 mL of the extract. A mixture of 3.5 mL of absolute ethanol and 0.3 mL of DPPH radical solution was used as a control. The radical scavenging activity (AOA %) was calculated using Eq. (4).

4 $$\:AOA\:\left(\%\right)=\:\left(1-\frac{{Abs}_{sample}-{Abs}_{blank}}{{Abs}_{control}}\right)\times\:100\:$$

#### Water-binding capacity

The water-binding capacity (WBC) of oyster mushroom stalk powder and chicken burger samples was determined according to Mounir^50^, with a slight modification. A 5 g sample was thoroughly mixed with 20 mL of distilled water in a pre-weighed 30-mL plastic centrifuge tube, and the mixture was allowed to stand for 45 min at room temperature (32 ± 2 °C). The sample was subsequently centrifuged at 960$\:\:\times\:\:$**g** for 30 min (centrifuge Model 3K15, motor (11133), SIGMA, Germany). The supernatant was carefully removed by decanting the sample, the tube was re-weighted, and the new mass of the sample was then recorded. The WBC was calculated using Eq. (5).

5 $$\:WBC\:\left(\%\right)=\frac{\:{W}_{2}-\:{W}_{1}}{\:{W}_{1}}\times\:100$$

Where: W_1_ and W_2_ are the weights of the sample before and after the centrifuge, respectively.

#### Cooking quality

The cooking loss and shrinkage were determined according to Polizer et al.^51^ using Eqs. (6) and (7).

6 $$\:Cooking\:loss\:\left(\%\right)=\left(\frac{{W}_{1}-{W}_{2}}{{W}_{1}}\right)\times\:100$$

7 $$\:Shrinkage\:\left(\%\right)=\:\left(\frac{{D}_{1}-\:{D}_{2}}{{D}_{1}}\right)\times\:100$$

Where W_1_ and D_1_ are the weight (g) and diameter (mm) of the raw burger sample, respectively, and W_2_ and D_2_ are the weight (g) and diameter (mm) of the cooked burger sample, respectively.

#### Color quantification

The color of oyster mushroom stalk powder and raw chicken burgers was determined according to Mounir et al.^52,53^ using a Hunter’s Lab color analyzer (Color Flex EZ Spectrophotometer, USA), which was calibrated with a black and white standard tile using the L* a* b* scale before measurement. Samples were placed in the standard cup, and the color values of the sample were recorded as L*, a*, and b*.

The total color change (*ΔE*) was calculated according to Vega-Gálvez et al.^54^ using Eq. (8), while the browning index (BI) was determined according to Shittu et al.^55^ using Eq. (9).

8 $$\:\varDelta\:E=\sqrt{{{\varDelta\:L}^{*}}^{2}{{+\varDelta\:a}^{*}}^{2}+\varDelta\:{{b}^{*}}^{2}}$$

9 $$\:BI=\:\frac{100\:\left(x\:-0.31\right)}{0.17}$$

Where $$\:x=\:\frac{{a}^{*}\:+1.75\:{L}^{*}}{5.645\:{L}^{*\:}+\:{a}^{*}-\:3.012\:{b}^{*}}$$

#### Texture profile analysis

The textural characteristics of different raw burger samples, including hardness (N), adhesiveness (g.cm), cohesiveness, resilience, springiness (mm), and chewiness (g cm), were determined using a texture analyzer (Brookfield CT3 texture analyzer, USA), according to Bourne^56^, with slight modifications. This analysis involves two-cycle compression of the sample to 50% of its original height using a cylindrical probe (TA-AACC36) equipped with a 10-kg load cell at a constant test speed of 2 mm/s.

The shear force (N) of different cooked burger samples was also determined using a texture analyzer (Brookfield, CT3 texture analyzer, USA). This analysis involves the sample rupture using a fixture (TA-SBA) equipped with a 10-kg load cell at a constant test speed of 3 mm/s.

### Statistical analysis

The obtained results were presented as the mean value ± standard deviation (SD) of three replicates and statistically analyzed using one-way ANOVA^57^ combined with Duncan’s multiple-range test^58^ to identify the significant differences between the response variables at *P* ≤ 0.05. Additionally, a correlation between the substitution level and different response variables was determined by computing Pearson’s correlation coefficient.

## Electronic supplementary material

Below is the link to the electronic supplementary material.

Supplementary Material 1

## Data Availability

All data generated or analyzed during this study are included in this published article.

## Abbreviations

AD: Adhesiveness (g.cm)
AOA: Antioxidant activity (%)
BI: Browning index
CH: Chewiness (g.cm)
CL: Cooking loss (%)
CO: Cohesiveness
ΔE: Total color change
H: Hardness (N)
SF: Shear force (N)
SH: Shrinkage (%)
SP: Springiness (mm)
TFC: Total flavonoid content (mg QE/g db)
TPC: Total phenolic content (mg GAE/g db)
WBC: Water-binding capacity (%)
