# Supplementary material for: Assessing the effects of partially substituting chicken breast meat with oyster mushroom stalk powder on the quality attributes of mushroom-chicken burgers
Source: Sci Rep. 2025 Feb 5;15:4361. doi: 10.1038/s41598-025-86127-3 (PMC11799463; doi:10.1038/s41598-025-86127-3)
Supplement: Supplementary file 1 — Supplementary Material 1 [file 41598_2025_86127_MOESM1_ESM.docx]

| Ingredients (g) | Control sample | Mushroom-chicken burgers | | | |
| --- | --- | --- | --- | --- | --- |
|  | 0.00 | 2.5% | 5% | 7.5% | 10% |
| Chicken breast meat | 500 | 487.5 | 475 | 462.5 | 450 |
| Mushroom stalk powder | 0.00 | 12.5 | 25 | 37.5 | 50 |
| Ground onion | 20 | 20 | 20 | 20 | 20 |
| Egg | One egg | One egg | One egg | One egg | One egg |
| Salt | 3 | 3 | 3 | 3 | 3 |
| Spice mixture | 5 | 5 | 5 | 5 | 5 |

*Table S1 The different formulas used in the preparation of chicken burger samples*
